# *Seseli foliosum* (Somm. et Levier) Manden.—A Comprehensive Phytochemical and Biological Evaluation

**DOI:** 10.3390/molecules30030725

**Published:** 2025-02-05

**Authors:** Mariam Nersezashvili, Dali Berashvili, Malkhaz Jokhadze, Mariam Metreveli, Łukasz Świątek, Kinga Salwa, Łukasz Pecio, Krzysztof Kamil Wojtanowski, Adrianna Skiba, Izabela Korona-Głowniak, Gökhan Zengin, Krystyna Skalicka-Woźniak

**Affiliations:** 1 Direction of Pharmacognosy and Pharmaceutical Botany, Faculty of Pharmacy, Tbilisi State Medical University, Tbilisi 0186, Georgia; m.nersezashvili@tsmu.edu (M.N.); d.berashvili@tsmu.edu (D.B.); m.jokhadze@tsmu.edu (M.J.); 2 Institute of Phytopathology and Biodiversity, Batumi Shota Rustaveli State University, Batumi 6010, Georgia; metreveli.mariam@bsu.edu.ge; 3 Department of Virology with Viral Diagnostics Laboratory, Medical University of Lublin, 20-093 Lublin, Poland; lukasz.swiatek@umlub.pl (Ł.Ś.); kinga.salwa@umlub.pl (K.S.); 4 Department of Natural Products Chemistry, Medical University of Lublin, 20-093 Lublin, Poland; lukasz.pecio@umlub.pl (Ł.P.); adrianna.skiba@umlub.pl (A.S.); 5 Department of Phytochemistry, Institute of Soil Science and Plant Cultivation—State Research Institute, 24-100 Puławy, Poland; 6 Department of Pharmacognosy with Medicinal Plant Garden, Medical University of Lublin, 20-093 Lublin, Poland; krzysztofkamilw@gmail.com; 7 Department of Pharmaceutical Microbiology, Medical University of Lublin, 20-093 Lublin, Poland; izabela.korona-glowniak@umlub.pl; 8 Department of Biology, Faculty of Science, Selcuk University, Konya 42130, Turkey; gokhanzengin@selcuk.edu.tr

**Keywords:** coumarin, volatiles, bacteria, fungi, cytotoxicity, antioxidant, anti-enzymatic, chemistry, centrifugal partition chromatography CPC

## Abstract

The genus *Seseli* L. (Apiaceae family) is widespread across Europe and Asia, with ten species identified in Georgia. Among these, *Seseli foliosum* (Somm. et Levier) Manden., is notable for its unique pharmacological properties. To our knowledge, comprehensive phytochemical and biological investigations have not yet been conducted. The primary aim of this research is to explore the chemical and biological properties of *S. foliosum*, thereby enhancing its potential applications in medicine and related fields. Different chromatographic techniques were utilized to isolate individual compounds and to identify the chemical composition of *S. foliosum* MeOH and Et_2_O extracts from seeds and roots. A battery of biological assays (antimicrobial, antioxidant, enzymatic, anxiolytic, and cytotoxic) were employed to assess the pharmacological properties of the extracts. The results from gas chromatography with mass spectrometry (GC/MS) revealed that both MeOH and Et_2_O extracts contain a diverse array of compounds, including monoterpenoids, sesquiterpenoids, and phenolic compounds. Furanocoumarin edultin was isolated from the MeOH extract by liquid–liquid separation (LLS). The MeOH extracts exhibited important antioxidant, enzyme inhibitory, and antimicrobial activities with notable efficacy against *Staphylococcus aureus* (MIC 125 µg/mL) and *Candida glabrata* (MIC 62.5 µg/mL). Underground Et_2_O extracts showed advanced cytotoxic activity, particularly against hypopharyngeal carcinoma cells (CC_50_ 22.33 µg/mL and 27.16 µg/mL, respectively). The study provides a wide-range analysis of the phytochemical composition and biological activities of *S. foliosum*, highlighting its potential as a source of bioactive compounds. These findings contribute to the understanding of the therapeutic potential of *S. foliosum* and lay the groundwork for further pharmacological and clinical research.

## 1. Introduction

The Apiaceae, formerly known as the Umbelliferae, represents one of the most widespread plant families globally. Numerous species within the Apiaceae are utilized extensively as vegetables, spices, and medicinal plants. This family encompasses approximately 450 genera and 3700 species worldwide. In Georgia, there are approximately 70 genera and about 180 species belonging to this family. Of these, 58 species are endemic, with 21 species being endemic to Georgia and 37 species endemic to the Caucasus region [1]. The genus *Seseli* L. belongs to the Apiaceae family and is widespread across Europe and Asia. Its distribution area includes the Iranian-Turkish, Euro-Siberian, and Eastern Mediterranean regions. In Georgia, there are twelve species of this genus. Among these is *S. foliosum* (Somm. et Levier) Manden., which is also known by its synonyms *Silaum foliosum* (Sommier & Levier) Grossh. and *Silaus foliosus* Sommier & Levier. The local name for *Seseli* is “Sasuqa”. Additionally, the pronunciations “Sezeli” or “Zezeli” are also accepted among Georgian scientists. The Georgian scientific name for *S. foliosum* is “Shefotlili Seseli” [2]. *S. foliosum* has a Transcaucasian distribution and is believed to spread along the Georgian–Turkish boundary. Some sources assert that *S. foliosum* is endemic to the Adjara-Lazeti region. Adjara is a region in Georgia located in the southwestern part of the country, while Lazeti is a region situated within the current boundaries of Turkey, with a partial extension into Georgia, historically part of southwestern Georgia [3]. *S. foliosum* is one of the unique species in Adjara, found exclusively in hemixerophilic habitats on forest cliffs at elevations ranging from 500 to 1000 m above sea level [4]. In Georgia, the plant grows wild on steep slopes and between rocks, making its collection particularly challenging for researchers [2].

The antioxidant properties of plant-derived products have emerged as a critical tool in evaluating their efficacy as therapeutic agents. The plant kingdom offers a diverse array of secondary metabolites that act as natural antioxidants [5]. Numerous studies have demonstrated an opposite relationship between the consumption of fruits and vegetables and the risk of developing chronic diseases. These health benefits are largely attributed to the presence of antioxidant phytochemicals in these foods. Phytochemicals, widely distributed in various foods and medicinal plants, play an essential role in preventing and managing chronic diseases induced by oxidative stress (OS) by acting as antioxidants [6]. OS, arising from an imbalance between reactive oxygen species (ROS) production and the body’s detoxification capacity, is a key pathogenic factor in chronic diseases such as diabetes, inflammatory disorders, cardiovascular diseases, cancer, cataracts, autism, and aging [7]. It is also a well-documented pathological hallmark of neurodegenerative diseases (NDDs). Although synthetic antioxidants have been widely employed to inhibit lipid oxidation in the food industry, their pharmacological applications are restricted due to toxicological concerns [8]. Conversely, plants possess an intrinsic ability to biosynthesize a broad spectrum of non-enzymatic antioxidants, which mitigate ROS-induced oxidative damage. Consequently, plant-derived antioxidant molecules and their associated defense mechanisms offer significant potential for preventing various diseases.

The improper and excessive use of antibiotics has significantly accelerated the emergence and spread of bacterial resistance, particularly among multidrug-resistant (MDR) and extensively drug-resistant (XDR) bacteria [9]. Among drug-resistant pathogens, *Staphylococcus aureus* is a leading cause of both hospital-acquired and community-acquired infections, capable of inducing a range of site-specific infections. One of the most severe forms is *S. aureus* bacteremia (SaB), which is associated with a high mortality rate of approximately 25% [9]. A study reported that in 2019, bacterial antimicrobial resistance (AMR) accounted for approximately 1.27 million deaths globally. Another significant opportunistic pathogen is *Staphylococcus epidermidis*, a commensal organism found on human skin and mucous membranes. When introduced into the body, *S. epidermidis* can form biofilms, enabling colonization and shielding the bacteria from immune responses and antibiotic treatments. This contributes to persistent and challenging infections. Furthermore, recent research has raised interest in the potential role of *S. epidermidis* biofilms in breast oncogenesis [10]. Similarly, the fungal microbe *Candida glabrata* has emerged as an opportunistic pathogen, now ranking as the second-leading cause of candidiasis in Europe and North America. *C. glabrata* is intrinsically resistant to antifungal drugs, exhibits remarkable metabolic adaptability, and can evade immune system detection [11]. The increasing prevalence of MDR and XDR infections underscores the urgent need for pharmaceutical industries to prioritize the development of new antibiotics to combat these threats effectively.

Globally, cancers of the head and neck (HNC), as well as the gastrointestinal (GI) tract, are among the most common malignancies, exhibiting high rates of morbidity and mortality [12]. The development of GI cancers is closely linked to many underlying risk factors, including but not limited to obesity, unhealthy diet, and physical inactivity [13]. Furthermore, both GI cancers and HNC have a significant association with tobacco smoking and alcohol consumption. Interestingly, the HNCs are often associated with infectious agents, primarily oncogenic human papillomaviruses [14]. At the same time, for GI cancers, such a relation is observed mainly in the case of primary hepatocellular carcinomas, which are linked to infection with hepatotropic viruses, mostly hepatitis B and C viruses [13]. Although novel chemotherapy and immunotherapy have demonstrated substantial improvement in the treatment of HNC and GI cancers, there is still a considerable need to develop new strategies for treating and preventing these diseases [12]. That is why, as a part of our studies, we have evaluated the anticancer potential of *S. foliosum* extracts on cells representing the HNC and GI cancers. The non-cancerous Vero cells were used as a reference for cytotoxicity studies.

Anxiety-related disorders are among the most prevalent psychiatric conditions affecting humans. Anxiety is a complex mental illness characterized by persistent, overwhelming concerns and unwarranted apprehension. The global prevalence of anxiety disorders ranges from 7.3% to 28.0%, highlighting their significance as a worldwide health concern [15]. Stress is one of the key factors contributing to the development of anxiety disorders. Despite extensive research involving model organisms, in vitro studies, and clinical trials, effective treatments for anxiety remain insufficient [16]. The current range of anxiolytic medications struggles to adequately address the needs of the large population affected by these disorders [17]. Moreover, despite benzodiazepines and other clinically approved drugs—such as barbiturates, gabapentin, pregabalin, valproate, vigabatrin, and tiagabine—are commonly employed in the treatment of anxiety disorders, their anxiolytic effects are often secondary rather than primary [18]. Furthermore, these drugs, particularly benzodiazepines, are subject to strict regulatory controls due to their potential for addiction. In addition to these challenges, current anxiolytic therapies are associated with numerous side effects, including decreased psychomotor activity, impairments in verbal learning and memory processes, and the development of pharmacological tolerance [19]. As a result, the development of novel therapies, particularly those with improved safety profiles that can serve as alternatives to synthetic drugs, remains a central focus in the ongoing research and development of psychotropic medications targeting anxiety disorders.

Phytochemical studies indicate that species within the genus *Seseli* predominantly contain coumarins, flavonoids, essential oils, and terpenoids. Coumarins are present in nearly all species of the genus *Seseli*. In 1985, Chubinidze et al. [20] isolated four simple coumarins—osthole, suberosin, umbelliferone, and scopoletin—and one furanocoumarin, edultin, from various parts of *S. foliosum* growing in Georgia. Specifically, suberosin, osthole, and edultin were isolated from the roots, scopoletin from the seeds, and both scopoletin and umbelliferone from the aerial part of the plant. To our knowledge, this is the only publication describing the chemical composition of *S. foliosum*. Some species of the genus *Seseli* are used in the treatment of central nervous system (CNS) disorders such as anxiety and epilepsy [21]. Additionally, antibacterial, antifungal, antitumor, anti-inflammatory, antinociceptive, and anxiolytic effects have been confirmed for various *Seseli* species [21]. However, our team was unable to find any studies describing the biological activity of *S. foliosum*. In addition, to the best of the authors’ knowledge, since 1985, neither of the modern instrumental methodologies has been used to extend knowledge regarding the phytochemical profile of *S. foliosum*, while biological studies have not been conducted at all.

Given the growing global interest in plant-derived antioxidants and bioactive compounds, *S. foliosum* offers a promising avenue for the discovery of natural products with therapeutic applications. This research aimed to conduct a comprehensive phytochemical and biological investigation of *S. foliosum* collected in Georgia. For this purpose, advanced chromatographic techniques were used and let to reveal diverse secondary metabolites. Biological activities were assessed through antioxidant, antimicrobial, and cytotoxic assays. Our findings represent the first comprehensive report on the biological profile and updated chemical composition of *S. foliosum*, highlighting its potential as a source of bioactive compounds with therapeutic applications. These results make *S. foliosum* a valuable subject for further exploration, particularly in the context of addressing issues such as oxidative stress-related disorders, antibiotic resistance, and cancer.

## 2. Results and Discussion

### 2.1. Extract Analyses, Isolation of Coumarin and Structural Elucidation of Isolate

Preliminary analyses based on high-performance liquid chromatography with diode array detector (HPLC-DAD) confirmed the presence of coumarins in both the seed and root MeOH extracts of *S. foliosum*. Figure S1 (Supplementary Materials) shows the HPLC-DAD chromatogram of *S. foliosum* seed MeOH (SfSMeOH) extract.

To identify the major peak in the extract, a combination of HPLC-DAD and HPLC-ESI-QTOF-MS/MS techniques was employed. The high-resolution QTOF-MS/MS was used to analyze the fragmentation pattern of the selected compound in the SfSMeOH extract, which allowed us to identify the skeleton of benzopsoralen with molecular ion [*m*/*z*] ([M+Na]^+^) 409.1256 and following MS/MS fragments [*m*/*z*] 309.0745, 249.0518, 227.0704. The furanocoumarin was identified as edultin (Figure S2).

Edultin (8(S),9(R)-9-angeloyloxy-8,9-dihydrooroselol) showed excellent antitumor-promoter activity. In addition, edultin exhibits average antibacterial activity towards *Pseudomonas aeruginosa* and *S. aureus* [22]. Thus, it became the compound of interest, and the aim was to elaborate an efficient isolation technique. To optimize the isolation process, partition coefficient (*Pi*) values were determined through shake-flask experiments. These values, ideally falling between 0.4 and 2.5, were used to select the most suitable biphasic solvent systems for liquid–liquid separation (LLS).

For edultin, the solvent system P20 (2:1:2:1, *v*/*v*/*v*/*v*) was identified as the most effective, successfully isolating the compound (*Pi* 0.6). The yield was 27 mg edultin out of 60 mg/mL extract, with a purity of approximately 95% (the same solvent system let us isolate the same compound from *S. foliosum* root MeOH extract (SfRMeOH) as well, but with the less purity (around 80%) and the further purification was necessary).

**Figure 1:**
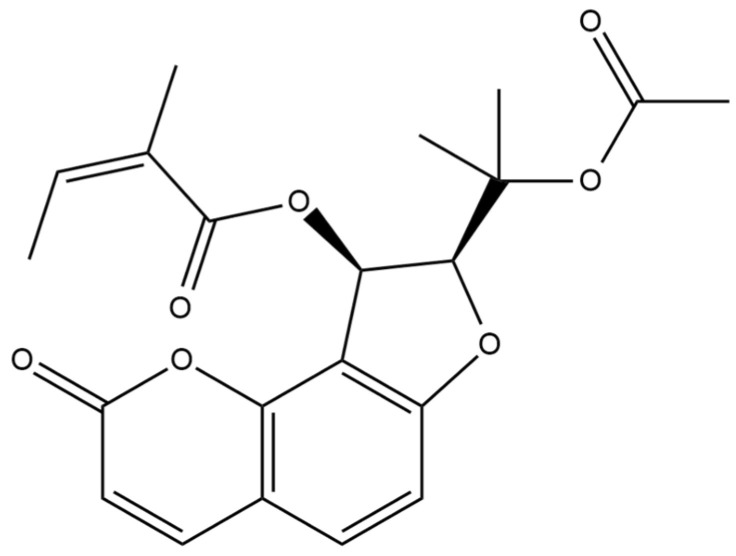
Chemical structure of edultin.

The chemical structure of the isolated coumarin was confirmed using 1D- and 2D- NMR. The spectroscopic data obtained matched those documented in spectral libraries and literature references, thereby validating the successful isolation and identification of the target compound—edultin (Figure 1).

The compound showed signals in its ^1^H and ^13^C NMR spectra assignable to the oroselol diester (furanocoumarin-type) with an acetyl group located at C-2′ and an angeloyl group at C-3′. The coupling constant of H-2′ and H-3′ (*J* = 6.8 Hz) indicated that their relative configuration was cis (Table S1, Figure S3) [23].

The successful isolation and characterization of coumarins, particularly furocoumarin edultin, from *S. foliosum* seeds underscore the plant’s potential as a source of bioactive compounds. The ability to isolate this compound in high quantities and purities suggests that *S. foliosum* could be a valuable resource for developing natural products with therapeutic applications. In addition, the high purity level of the isolated compound indicates the effectiveness of the LLS method. This opens new avenues for further pharmacological studies to explore the full spectrum of biological activities of this compound.

### 2.2. Gas Chromatography with Mass Spectrometry Results

Alkanes were used as standards to compute the retention indices (RI). By comparing the retention times (RT), mass spectra, and RI of the essential oils to those described in the literature (NIST 20 and Wiley Libraries) and MS libraries (Wiley), the chemical components of the volatiles were identified. Traditional library searches just compare spectra rather than taking retention parameters into account. In this study, libraries were searched using a combination of storage indexes, which made compound identification simpler and more accurate [23]. The device’s retention index spectrum libraries were also utilized in this study. The same analytical procedure as the identical column provided in the library was applied for better results.

The analysis conducted via GC/MS led to the identification of distinct compounds in the MeOH and Et_2_O extracts of *S. foliosum*. Table 1 gives the list of the most abundant volatiles that have been identified in seed and root MeOH and Et_2_O extracts, respectively.

Essential oils are widely recognized for their therapeutic properties. Notably, the identified compounds were predominantly monoterpenoids, sesquiterpenoids, and phenolic compounds, as well as fatty acids. The presence of these terpenoids is particularly intriguing given their well-documented bioactivities, including antimicrobial, anti-inflammatory, and antioxidant properties.

Terpenoids have shown great promise as potential therapeutic agents for treating depression and anxiety disorders. Compounds such as *β*-caryophyllene, linalool, caryophyllene oxide, and *p*-cymene have been extensively studied for their pharmacological properties, including neuroprotection, anti-inflammatory effects, antibacterial activity, neurotransmitter regulation, and antioxidant capabilities. Preclinical evidence highlights their diverse mechanisms of action, supporting their potential as antidepressant and anxiolytic agents [28]. For example, *β*-caryophyllene and linalool have demonstrated high binding affinities to acetylcholinesterase (AChE), enhancing neurotoxic effects [29]. Linalool also exhibits antimicrobial, antioxidant, antiproliferative, anti-angiogenesis, and anti-metastasis activities [30]. A study by Gunaseelan et al. [31] showed that linalool effectively suppressed non-melanoma skin cancer (NMSC) in mice exposed to chronic ultraviolet B (UVB) radiation. Pretreatment with linalool significantly reduced the expression of proliferation markers, including nuclear factor kappa-light-chain-enhancer of activated B cells (NF-κB), tumor necrosis factor-alpha (TNF-α), interleukin-6 (IL-6), cyclooxygenase-2 (COX-2), vascular endothelial growth factor (VEGF), transforming growth factor-beta1 (TGF-β1), and B-cell lymphoma 2 (Bcl-2). Histopathological analysis confirmed that linalool prevented dysplasia and squamous cell carcinoma (SCC) development caused by UVB radiation exposure through both topical application and intraperitoneal injection.

Isoborneol has been found to enhance intestinal drug absorption by bidirectionally regulating P-glycoprotein [32]. It exhibits antimicrobial activity against *Escherichia coli*, *S. aureus*, and *Mucor* sp. while also showing anti-inflammatory, analgesic, antipyretic, antibacterial, neuroprotective, and permeation-promoting effects [32]. Phytol is emerging as a dietary compound for cancer prevention, with evidence showing that it alters carcinogenesis pathways, such as mitochondrial dysfunction, oxidative damage, intracellular Ca^2^⁺ deregulation, and histone deacetylation, at physiological concentrations (≤10 μM) [33]. Limonene has shown significant DPPH (1,1-diphenyl-2-picrylhydrazyl) radical scavenging activity and mild ABTS (2,2′-azino-bis(3-ethylbenzothiazoline) 6-sulfonic acid) effects, whereas α-pinene has exhibited moderate DPPH activity with lower ABTS effects [34].

Safranal has demonstrated anti-inflammatory, antidepressant, anxiolytic, antiasthmatic, antihypertensive, anticonvulsant, anticancer, antitussive, and antigenotoxic effects. It has garnered significant interest for its antioxidant benefits for human health. Safranal exhibits antidiabetic effects by strengthening the antioxidant defense system and mitigating oxidative stress in diabetic models, suggesting its potential for managing diabetes and related complications. Additionally, safranal has been shown to have positive effects on anxiety, depression, epilepsy, and memory disorders, with ongoing research investigating its antipsychotic properties [35].

Sesquiterpenoids, the largest subgroup of terpenoids, have numerous applications in pharmaceuticals, as well as in the flavor and fragrance industries. Bergamotene, for example, demonstrates antioxidant, anti-inflammatory, immunosuppressive, cytotoxic, antimicrobial, antidiabetic, and insecticidal activities [36]. Caryophyllane sesquiterpenes, particularly caryophyllene oxide, are prominent for their antibacterial, antifungal, genoprotective, antioxidant, anti-inflammatory, chemosensitizing, and antiproliferative effects. These compounds exhibit significant chemopreventive potential, as they block carcinogen-mediated deoxyribonucleic acid (DNA) damage and protect non-cancerous cells from drug toxicity while also showing antiproliferative and chemosensitizing activities against cancer cells [37]. In a study by Kamikubo et al. [38], *β*-caryophyllene was found to inhibit lipid accumulation induced by palmitic acid in human liver cancer cells (hepatocellular carcinoma G2, HepG2), indicating its potential for preventing non-alcoholic fatty liver disease (NAFLD) and related metabolic disorders. Ullah et al. [39] also demonstrated *β*-caryophyllene’s antioxidant and antibacterial potential as a food packaging agent. Zein/polycaprolactone nanofibers loaded with *β*-caryophyllene and halloysite enabled sustained release, showing strong DPPH scavenging and effectiveness against *Bacillus subtilis* and *E. coli*. *β*-caryophyllene also exhibits anti-inflammatory and anti-ulcer properties.

Fatty acids such as linoleic acid, oleic acid, and palmitic acid have also gained attention for their pharmacological properties. Higher intakes of linoleic acid are associated with improved cardiometabolic health, as it lowers low-density lipoprotein (LDL) cholesterol and reduces the risk of coronary heart disease, stroke, and type-2 diabetes [40]. Oleic acid, a natural ligand of peroxisome proliferator-activated receptor gamma (PPARγ), promotes adipocyte differentiation by regulating gene expression, enhancing its therapeutic potential in metabolic disorders [41]. Palmitic acid, which constitutes 20–30% of the human body’s fatty acids, serves as an energy source and plays a role in cell membrane structure and function. Modern studies show that palmitic acid exhibits anti-inflammatory, antioxidant, and immune-enhancing effects, and its antitumor activity targets cancers such as gastric, liver, cervical, breast, and colorectal cancer. Its mechanisms include apoptosis induction, inhibition of tumor proliferation and metastasis, and enhancement of chemotherapy sensitivity through ROS generation [42].

Phenolic compounds, predominantly coumarins, such as isopsoralen, 5′-isopropenylangelicin, methoxsalen, and osthole were also identified. The mentioned coumarins are well known for their diverse pharmacological effects, including anticoagulant, anti-inflammatory, and anticancer activities.

Further research is warranted to explore the biological activities of the identified compounds and to evaluate the efficacy and safety of *S. foliosum* extracts in various applications. Additionally, understanding the ecological roles of these compounds in *S. foliosum* could provide insights into the plant’s adaptive strategies and interactions with its environment.

### 2.3. Phenolic Compounds

Total phenolic content (TPC) has been investigated across various *Seseli* species. According to Zengin et al. [21], methanol and water extracts of *S. gummiferum* Pall. ex Sm. and *S. transcaucasicum* (Schischk.) contain meaningful amounts of phenols, ranging from 19.09 to 24.33 mg GAE/g, respectively. The same study highlights that methanolic extracts of *S. pallasii* Besser, *S. libanotis* (L.) Koch ssp. *libanotis*, and *S. libanotis* (L.) Koch ssp. *intermedium* (Rupr.) P. W. Ball exhibits higher TPC levels, ranging from 84.04 to 87.53 mg GAE/g, as reported by Matejic et al. [43].

The total phenolic content was quantified by conversion to gallic acid equivalents (GAE). As shown in Table 2, the SfSMeOH contained a great number of phenolic compounds compared to the roots. Specifically, the seed methanol extract yielded significantly higher levels of phenolic components than the root extract (19.27 mg GAE/g and 10.41 mg GAE/g, respectively). The finding can be explained by various factors. For example, phenolics can protect against oxidative stress during dormancy and germination processes. Similarly, several researchers reported higher levels of phenolics in several plant seeds as compared to other plant parts, including roots [44].

### 2.4. Antioxidant and Anti-Enzymatic Activity

In the phosphomolybdenum assay, the highest antioxidant capacity was observed in the SfRMeOH extract (1.23 µmol TE/g). However, the SfSMeOH exhibited nearly equivalent antioxidant activity (1.19 µmol TE/g). Both extracts demonstrated significant radical scavenging potential (DPPH: 10.76–37.97 mg TE/g; ABTS: 14.12–28.94 mg TE/g). Notably, the seed extract displayed markedly higher scavenging potential compared to the root extract. In terms of reducing activity measured by CUPRAC and FRAP assays, the SfSMeOH showed nearly double the reducing potential compared to the root extract (CUPRAC: 56.18–28.87 mg TE/g; FRAP: 16.05–35.86 mg TE/g). Similarly, the metal chelating assay indicated higher metal chelating potential in the SfSMeOH extract compared to the SfRMeOH extract (27.51 mg EDTAE/g and 19.97 mg EDTAE/g, respectively). Comprehensive results from all antioxidant assays are presented in Table 3.

The high antioxidant activity observed in SfSMeOH and SfRMeOH is likely attributed to their substantial content of polyphenolic compounds. Previous studies have highlighted the notable antioxidant properties of *Seseli* species. Önder et al. [5] evaluated the antioxidant activity of MeOH and ethyl acetate (EtOAc) extracts derived from the aerial parts of various *Seseli* species growing in Turkey. The results demonstrated remarkable DPPH radical scavenging activity in these species. For example, the EtOAc extract of *S. peucedanoides* exhibited a radical scavenging activity with an IC_50_ value of 0.49 mg/mL. Similarly, the MeOH extracts of *S. resinosum*, *S. gummiferum*, and *S. peucedanoides* showed significant antioxidant activity with IC_50_ values of 0.086 mg/mL, 0.088 mg/mL, and 0.091 mg/mL, respectively. The strongest DPPH radical scavenging activities were observed in the EtOAc extracts of *S. peucedanoides* (IC_50_ = 0.49 mg/mL) and *S. libanotis* (IC_50_ = 0.75 mg/mL) [5]. The extract of *S. rigidum* also displayed noteworthy antioxidant activity, ranging from 12.99 to 9.87 g/mL trolox equivalents [45]. Additionally, studies on the Algerian *S. tortuosum* revealed moderate antioxidant potential. However, this species exhibited a significantly stronger anti-Alzheimer effect on butyrylcholinesterase (BChE) with IC_50_ values of 9.14 ± 1.74 μg/mL and 34.75 ± 1.99 μg/mL, respectively [46]. In a study by Zengin et al. [21], *S. gummiferum* and *S. transcaucasicum* also exhibited notable antioxidant activity across all assays (DPPH: 5.51–11.45 mg TE/g; ABTS: 43.46–51.91 mg TE/g; CUPRAC: 41.67–53.20 mg TE/g; FRAP: 31.26–34.14 mg TE/g; MCA: ranging from 14.38 to 38.57 mg EDTAE/g; PDA: 0.66–1.18 mmol TE/g, respectively).

The utilization of enzyme inhibitors has become a prevalent strategy in drug discovery within pharmacology. Enzymes are implicated in a broad spectrum of human diseases, prompting the development of specific inhibitors aimed at suppressing their activity and serving as therapeutic agents [47].

Enzyme inhibitors are now widely employed in pharmacology for drug discovery. Given enzymes’ involvement in numerous human diseases, various specific inhibitors have been developed to target their activity, thereby serving as therapeutic agents [47].

The enzyme cholinesterase plays a crucial role in Alzheimer’s disease and is thus a primary therapeutic target. Tyrosinase, a copper-containing enzyme, regulates melanogenesis, making its inhibition a popular approach for managing skin pigmentation. α-glucosidase and α-amylase are enzymes involved in carbohydrate digestion, and their inhibition can help regulate postprandial glucose levels in the body, thereby controlling blood glucose levels in patients with type 2 diabetes [48]. Hence, the study holds promise for investigating neurodegenerative diseases, antimelanogenesis, and antidiabetic activities in future research.

Table 4 reveals that both extracts demonstrate ACHE and BCHE inhibitory activity. The SfRMeOH exhibits slightly higher ACHE inhibitory potential compared to the SfSMeOH (2.79–2.70 mg GALAE/g), whereas the SfSMeOH displays higher BCHE inhibitory activity (2.27 mg GALAE/g) compared to the SfRMeOH (1.73 mg GALAE/g). Both extracts exhibit moderate anti-tyrosinase activity, with the SfSMeOH showing higher potency (36.74 mg KAE/g). Considering that kojic acid is a phenolic compound (4-pyron derivative), which acts as an inhibitor of tyrosinase by scavenging ROS and free radicals, particularly chelating the copper ions in the active site of tyrosinase, anti-tyrosinase activity may be attributed to the phenolic composition present in both extracts, particularly in the seeds. Additionally, both extracts demonstrate similar inhibitory effects against α-amylase and α-glucosidase. Acarbose, used as a reference to α-amylase and α-glucosidase inhibition tests, exhibits α-glucosidase and α-amylase inhibitory activity by reducing carbohydrate digestion and absorption, leading to better control of blood sugar levels, thus influencing blood glucose regulation and indirectly reduces OS [49]. It can be considered that *S. foliosum* might have the possibility to indirectly inhibit enzyme activity and reduce oxidative stress by managing glucose levels.

These findings provide further support to the proposition by Zengin et al. [21] that *Seseli* species hold potential as natural sources for the development of novel pharmaceuticals utilizing natural enzyme inhibitors.

### 2.5. Microbiological Evaluation

The antibacterial activity of *S. foliosum* MeOH and Et₂O extracts was assessed against several bacterial and fungal strains. In this study, the strength of extract bioactivity was defined according to the interpretation of O’Donnell et al. [50], where an MIC higher than 1000 µg/mL was considered indicative of no bioactivity. The results, detailed in Table 5, indicate that both SfSMeOH and SfRMeOH possess good (MIC in the range 26–125 µg/mL) or moderate (MIC in the range 125–500 µg/mL) antimicrobial properties against Gram-positive bacteria and yeasts, making them potential candidates for the development of natural antibacterial and antifungal agents.

SfSMeOH exhibited higher activity against the tested bacterial and fungal strains than SfRMeOH. Neither extract demonstrated bioactivity against the Gram-negative bacteria tested or the *E. faecalis* reference strain.SfSMeOH demonstrated bactericidal effects against *S. aureus* (MIC = 125 µg/mL, MBC/MIC = 2). Additionally, the seed extract exhibited bactericidal activity against *S. epidermidis* (MIC = 250 µg/mL, MBC/MIC = 2). These findings suggest that the seed extract has the potential not only to inhibit the growth of staphylococci but also to kill these bacteria, indicating its potential as a source for treating infections caused by these pathogens. SfRMeOH showed good activity (MIC = 62.5 µg/mL) but exhibited bacteriostatic effects against *Micrococcus luteus* (MBC/MIC = 8) and moderate bioactivity against tested staphylococci (MIC = 250 µg/mL). Only SfSMeOH displayed anti-candida activity. Moderate and fungicidal activity was observed against *Candida parapsilosis* (MFC/MIC = 1), along with good but fungistatic activity against *C. glabrata* (MFC/MIC = 8).

The Et_2_O extract from *S. foliosum* seeds (SfSV) demonstrated moderate antibacterial activity toward Gram-positive bacteria, particularly against *S. aureus* (MIC = 250 mg/L, MBC/MIC = 8). In contrast, the Et_2_O root extract (SfRV) showed good activity against *M. luteus* (MIC = 250 µg/mL, MBC/MIC = 8). However, neither extract exhibited any activity against yeasts.

Even though the extracts exhibited significantly lower activity compared to reference antibiotics, we propose that the antibacterial activity (against certain Gram-positive strains) and antifungal activity observed in SfSMeOH and SfRMeOH hold considerable promise for the development of natural antimicrobial agents. The effectiveness of these extracts against a variety of microbial strains, including *S. aureus*, *S. epidermidis*, *M. luteus*, *C. parapsilosis*, and *C. glabrata*, highlights the broad-spectrum potential of *S. foliosum* extracts.

It was acknowledged that crude extracts typically show higher MIC values (e.g., 250–1000 µg/mL), reflecting lower concentrations of active components compared to purified compounds or essential oils. Future research should focus on isolating and identifying the specific compounds responsible for these antimicrobial activities. Understanding the mechanisms of action of these compounds can provide deeper insights into their potential therapeutic applications. Additionally, in vivo studies are necessary to evaluate the safety and efficacy of *S. foliosum* extracts in animal models, paving the way for clinical trials.

Notable antibacterial (against some Gram-positive strains) and antifungal activities observed in SfSMeOH and SfRMeOH suggest that this plant holds considerable promise for developing natural antimicrobial agents. The effectiveness against a variety of microbial strains, including *S. aureus*, *S. epidermidis*, *M. luteus*, *C. parapsilosis*, and *C. glabrata*, underscores the broad-spectrum potential of *S. foliosum* extracts.

Future research should focus on isolating and identifying the specific compounds responsible for these antimicrobial activities. Understanding the mechanisms of action of these compounds can provide deeper insights into their potential therapeutic applications. Additionally, in vivo studies are necessary to evaluate the safety and efficacy of *S. foliosum* extracts in animal models, paving the way for clinical trials.

### 2.6. Cytotoxicity

In the presented research, the cytotoxicity was tested against a panel of cell lines. Vero, a mammalian cell line established from the kidney of the African green monkey (*Cercopithecus aethiops*), is not only extensively used in virology studies for culturing various viruses (herpesviruses, rabies, influenza, dengue, measles, polio, rubella, and corona viruses) and antiviral studies but has also been used in many other applications, including studying intracellular bacteria (e.g., *Rickettsia* spp.), and assessment of the effects of chemicals, bacterial and environmental toxins, and plant extracts on mammalian cells at the molecular level [51]. Also, these cells can be maintained and passaged without showing signs of senescence, providing reliable and repeatable results. Moreover, the Vero cells are endorsed by ISO experts as suitable for studying in vitro cytotoxicity, according to ISO 10993-5:2009 [52]. That is why Vero cells were selected as non-cancerous cells in this research. The anticancer potential was assessed against cell lines derived from oral and gastrointestinal cancers, namely hypopharyngeal, stomach, and colon carcinomas. FaDu cells are a part of the ATCC Head and Neck Cancer Panel (ATCC, TCP-1012), AGS cells belong to the Stomach (Gastric) Cancer Panel (TCP-1008), while RKO represents the Colon Cancer Panel 2 (TCP-1007). According to ATCC (https://www.atcc.org; access date: 15 January 2025), FaDu cells were derived in 1968 from a punch biopsy of a hypopharyngeal tumor removed from a 56-year-old patient with squamous cell carcinoma. RKO is a poorly differentiated colon carcinoma cell line, while AGS is a cell line exhibiting epithelial morphology isolated in 1979 from the stomach tissue of a 54-year-old patient with gastric adenocarcinoma. Table 6 presents the CC_50_ values obtained for *S. foliosum* MeOH and Et_2_O extracts.

**Figure 2:**
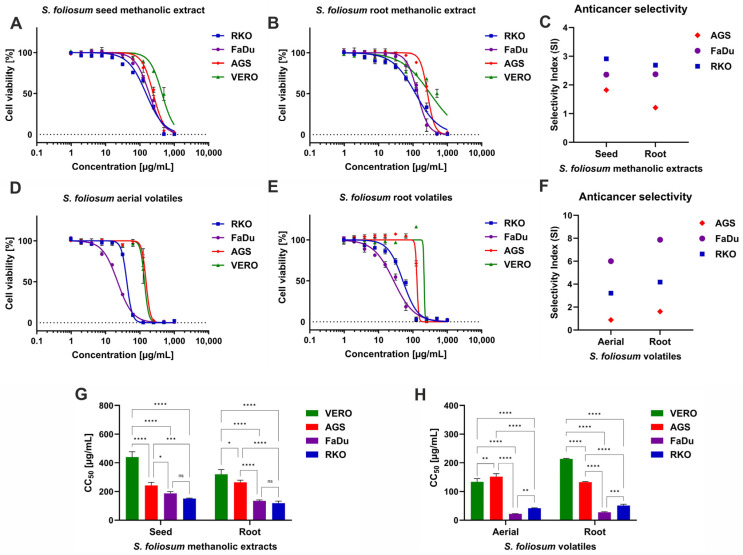
The cytotoxicity of *S. foliosum* methanolic extracts and volatiles (dose–response effects of SfSMeOH (**A**) and SfRMeOH (**B**); (**C**)—anticancer selectivity of *S. foliosum* MeOH extracts; dose–response effects of SfSV (**D**) and SfRV (**E**); (**F**)—anticancer selectivity of *S. foliosum* Et_2_O extracts; comparison of CC_50_ values with evaluation of statistical significance (two-way ANOVA with Tukey’s *post hoc* test) presented for *S. foliosum* MeOH extracts (**G**) and *S. foliosum* Et_2_O extracts (**H**), **** *p* < 0.0001, *** *p* < 0.001, ** *p* < 0.01, * *p* < 0.05, ns—not significant).

According to the previously described classification of the plant [48] extract cytotoxicity, the SfSMeOH, SfRMeOH, and SfRV showed weak cytotoxicity towards non-cancerous Vero cells. In contrast, SfHV showed moderate cytotoxicity, with a CC_50_ of 133.9 µg/mL. The analysis of dose–response curves presented in Figure 2A,B showed that SfSMeOH and SfRMeOH exerted higher cytotoxicity towards FaDu and RKO cells compared to Vero cells, and the selectivity index (SI) ranged from 2.36 to 2.91 (Figure 2C). The dose–response curves represent the relation between the concentration of tested extract and the viability of a particular cell line, expressed in the percentage of viable cells. Treatment with increasing concentrations of tested extracts resulted in a decrease in viability percentage. For example, the RKO cells treated with SfSMeOH in the concentration of 15.63 µg/mL showed viability of almost 95%, while at increasing concentrations of 31.25, 62.5, and 125 µg/mL, it was reduced to 85%, 75%, and 66%, respectively. Further increase in SfSMeOH concentration, at 250 µg/mL, decreased viability to 34%, while at 250 µg/mL, there were no viable RKO cells. As was shown in Figure 2G, there were no statistically significant differences (*p* > 0.05) between CC_50_ obtained for SfSMeOH or SfRMeOH extract on FaDu and RKO. Interestingly, the SfRV and SfSV showed even higher cytotoxicity, with the FaDu cell being the most sensitive (Figure 2D,E). The anticancer selectivity of SfSV and SfRV (Figure 2F) towards RKO was 3.21 and 4.18, respectively, while against FaDu was noticeably higher—6.0 and 7.87. For SfHV and SfRV, the obtained CC_50_ values against FaDu cells, 22.33 and 27.16 µg/mL, respectively, were the lowest among all tested samples, also significantly (*p* < 0.0001) lower than against non-cancerous cells (Figure 2H). The AGS cells derived from human gastric adenocarcinoma showed the lowest sensitivity to *S. foliosum* MeOH and Et_2_O extracts, with SI ranging between 0.88 and 1.82.

The American National Cancer Institute (NCI) suggests that significant anticancer activity for crude extracts should be reported when CC_50_ < 20 µg/mL after 48 h or 72 h incubation [53]. However, some authors consider results of CC_50_ below 30 µg/mL for crude extract as promising anticancer effects, worthy of further research [54]. Considering this, it can be concluded that *S. foliosum* aerial and root Et_2_O extracts show promising anticancer activity against cells originating from human hypopharyngeal carcinoma. Although we acknowledge that the chosen cancer panel is limited and does not represent all possible cancer cell lines from head and neck or gastrointestinal cancers, the collected data provided valuable insight into the anticancer potential of *S. foliosum*.

The selective anticancer potential of *S. foliosum* aerial and root Et_2_O extracts against FaDu and RKO cells is intriguing and requires further studies. Notably, the analysis of volatile compounds of *S. foliosum* tested herein showed that there are several compounds found only in Et_2_O extracts, namely *α*-pinocarvone, *D*-verbenone, lauraldehyde, globulol, palmitic acid, phytol, and linoleic acid. Thus, some of these compounds, alone or in combination, may be responsible for higher toxicity towards these specific cells. Little is known about the cytotoxicity of *D*-verbenone, a bicyclic ketone terpene. However, (1S)-(-)-Verbenone was reported to exert anti-inflammatory effects [55]. Its derivatives showed significant in vitro anticancer effects on human osteosarcoma cell lines (MG63 and Saos-2) [55] and breast cancer cell lines (MDA-MB231, MDA-MB453, MCF-7, and 4T1) [56] and significantly reduced tumor development in xenograft mouse models [55]. Globulol isolated from the fruits of *Eucalyptus globulus* Labill showed antifungal activity on *Alternaria solani*, *Fusarium graminearum*, *Rhizoctonia solani*, and *Venturia pirina* and antibacterial potential towards *Xanthomonas vesicatoria* and *Bacillus subtilis* [57]. *Eucalyptus citriodora* leaves essential oil was shown in vitro to inhibit SARS-CoV-2 replication in infected Vero-E6 cells, and further in silico studies revealed that globulol is probably responsible for this activity [58]. Unfortunately, no cytotoxicity of globulol on human or animal cell lines was reported in these studies. Phytol showed no significant cytotoxicity when incubated with mouse skin cells, followed by cell viability assessment with an MTT assay [59]. However, from a toxicological point of view, it is interesting that phytol used as a thinning agent in some vaping liquids containing cannabinoids inhaled by vaping pens might be responsible for severe pulmonary injury [60].

### 2.7. Anxiolytic Activity

Over the past five years, global anxiety levels have experienced notable fluctuations, influenced significantly by the COVID-19 pandemic. In the first year of the pandemic, the World Health Organization (WHO) reported a 25% increase in the global prevalence of anxiety and depression [61].

Georgia has faced significant challenges in addressing mental health issues, including anxiety disorders, during the last five years. The COVID-19 pandemic has exacerbated mental health challenges globally, and Georgia is no exception [62]. Thus, the importance of discovering new natural sources with anxiolytic activity has increased.

Based on previous research conducted by Widelski, which demonstrated the anxiolytic activity of *Seseli devenyense*, as well as ethnopharmacological studies documenting the traditional use of *Seseli tortuosum* in Iranian medicine for treating epilepsy and anxiety-related conditions [63], we decided to investigate the potential anxiolytic activity of Georgian *S. foliosum*.

To evaluate the effect of SfSMeOH and SfRMeOH on anxiety-like behavior, we utilized a larval zebrafish model. Five-day post-fertilization (5 dpf) zebrafish larvae were observed for their responses to a stressor, which was exposure to strong light. The fish’s locomotor activity and thigmotaxis were assessed during changing light–dark cycles.

**Figure 3:**
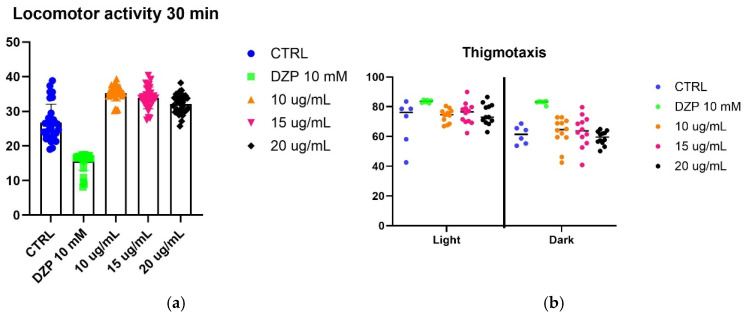

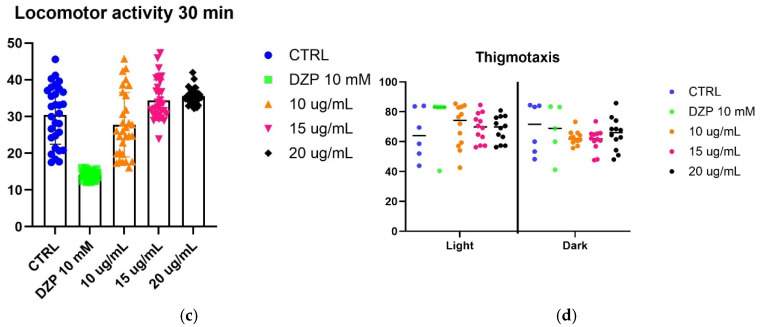
Effect of SfSMeOH and SfRMeOH on *Danio rerio* 5-dpf larvae locomotor activity. (**a**) Locomotor activity of SfRMeOH after 30 min; (**b**) thigmotaxis behavior of SfRMeOH; (**c**) locomotor activity of SfSMeOH after 30 min; (**d**) thigmotaxis behavior of SfSMeOH.

The assay did not reveal any anxiolytic activity in the extracts from *S. foliosum*. Considering that other extracts from the *Seseli* genus possess anxiolytic properties, it can be inferred that the observed lack of activity may be attributed to differences in the chemical composition of the tested extracts that may differ from those with reported activity. Alternatively, it is possible that compounds present in *S. foliosum* antagonize anxiolytic activity (results are presented in Figure 3).

Further studies focusing on the activity of fractions and pure compounds derived from *S. foliosum* could provide valuable insights into the pharmacological potential of the *Seseli* genus.

## 3. Materials and Methods

### 3.1. Chemicals

Analytical-grade methanol (MeOH), diethyl ether (Et_2_O), ethyl acetate (EtOAc), and *n*-hexane (Sigma-Aldrich, Darmstadt, Germany) were utilized for extraction and isolation processes. Chromatographic grade acetonitrile, ammonium formate, methanol, water, and formic acid (J.T. Baker, Deventer, The Netherlands) were employed for the HPLC-DAD and HPLC-ESI-QTOF-MS/MS analyses. Water was purified using a Simplicity^®^ water purification system (Millipore, Grenoble, France). DTNB (5,5-dithio-bis(2-nitrobenzoic) acid (Sigma, St. Louis, MO, USA), α-glucosidase (Saccharomyces cerevisiae, EC 3.2.1.20, Sigma), PNPG (4-N-trophenyl-α-D-glucopyranoside), Sigma)), AChE (acetylcholines-terase (electric ell acetylcholinesterase, type-VI-S, EC 3.1.1.7, Sigma)), BChE (butyrylcholinesterase (horse serum butyrylcholinesterase, EC 3.1.1.8, Sigma)) solution, acetylthiocholine iodide (ATCI, Sigma), butyrylthiocholine chloride (BTCl, Sigma), α-amylase solution (ex-porcine pancreas, EC 3.2.1.1), Sigma)) and tyrosinase solution (Sigma) was used for anti-enzymatic assays. Dimethyl sulfoxide (DMSO) for molecular biology (Sigma-Aldrich, Darmstadt, Germany). Minimal inhibitory concentration (MIC) of the tested extracts was evaluated for the panel of the reference microorganisms from the American Type Culture Collection (ATCC), including Gram-negative bacteria, Gram-positive bacteria, and fungi. The cytotoxicity of *S. foliosum* extracts was tested against non-cancerous Vero (ATCC: CCL-81, monkey kidney) cells and selected cell lines originating from cancers of the stomach (AGS; ATCC: CRL-1739, human gastric adenocarcinoma), hypopharynx (FaDu; ATCC: HTB-43, human hypopharyngeal squamous cell carcinoma), and colon (RKO; ATCC: CRL-2577, human colon cancer). The Vero cells were cultured in DMEM (Dulbecco’s Modified Eagle’s Medium; Corning, Tewksbury, MA, USA), and cancer cell lines were maintained in MEM (Minimum Essential Medium; Corning). Antibiotics (Penicillin–Streptomycin Solution, Corning) and fetal bovine serum (FBS; Corning) were added to all cell media. The MTT (3-(4,5-dimethylthiazol-2-yl)-2,5-diphenyltetrazolium bromide) was purchased from Sigma-Aldrich (St. Louis, MO, USA). Diazepam was purchased from Sigma-Aldrich, St. Louis, MO, USA). Phosphate-buffered saline (PBS) and trypsin were obtained from Corning, the sodium dodecyl sulphate (SDS) was from PanReac (PanReac AppliChem, Darmstadt, Germany), while dimethylformamide (DMF) was from Avantor (Avantor Performance Materials, Gliwice, Poland).

### 3.2. Plant Material

*Seseli foliosum* (Sommier & Levier) Manden. seeds and roots were collected during 2021 from the forest of the territory of Keda, Adjara, Georgia (Latitude: 41°36′0.00″ N; Longitude: 41°55′59.88″ E). A voucher specimen: BSU-FBI *Seseli foliosum* (Sommier. et Levier.) Manden.—№255. 5 September 2021 was deposited in Batumi State University. The plant material was identified by Dr. Mariam Metreveli (Batumi, Adjara, Georgia).

### 3.3. Statistical Analyses

Statistical significance was evaluated with GraphPad Prism (version 9.0.0 for cytotoxicity and version 10.3.1 for anxiolytic activity, Boston, MA, USA) using two-way ANOVA followed by Tukey’s post hoc test of significance, wherein *p* < 0.05 was considered statistically significant. All experiments were performed in triplicate.

### 3.4. Water-Bath Extraction

In total, 20 g of powdered *S. foliosum* seeds and roots were extracted separately with 200 mL pure MeOH. The procedure was repeated three times. Specimens were evaporated separately under vacuum; 4 g of dried extracts were received from both seeds and roots.

Additional seed and root extracts were obtained using maceration. Powdered seeds and roots were extracted via maceration with Et_2_O with a plant-to-solvent ratio of 1:10 g/mL for three days. The extracts were evaporated at a temperature of 25 °C. Finally, 0.2 g and 0.4 g of dried extracts were obtained from seeds and roots, respectively.

### 3.5. Liquid–Liquid Separation

Centrifugal partition chromatography (CPC) is a form of liquid–liquid separation that utilizes centrifugal force to maintain a liquid stationary phase, enabling efficient and high-purity separations of compounds based on their partitioning behavior between two immiscible liquids. CPC relies on the differential partitioning of solutes between two immiscible liquid phases within a pre-equilibrated biphasic solvent system, where one phase serves as the stationary phase, held in place by a centrifugal field, and the other functions as the mobile phase. CPC offers high selectivity and loading capacity by combining the principles of liquid–liquid extraction with chromatographic separation, while its resource-efficient design reduces solvent consumption, time, and labor costs by removing the need for expensive solid stationary phases or column packing procedures [64]. This technique accommodates raw samples containing compounds with a wide polarity range and has been successfully applied to isolate various natural products, including polyphenols, alkaloids, terpenoids, coumarins, proteins, and peptides, making it an indispensable tool in natural product research and pharmaceutical applications [64].

The methodology for liquid–liquid separation and HPLC-ESI-QTOF-MS/MS identification was developed based on the method described in Widelski et al. [65] with some appropriate modifications. The biphasic solvent system was selected by determining the partition coefficient (*Pi*) using the “shake-flask” method. Various ratios of the *n*-hexane/ethyl acetate/methanol/water (HEMWat) systems (6:5:6:5; 3:2:3:2; 2:1:2:1; 5:2:5:1; *v*/*v*/*v*/*v*) were tested to identify the most suitable solvent system for CPC.

Initially, the semi-preparative column was filled with the stationary upper phase. The rotational speed was then set to 1700 rpm, and the mobile lower phase was pumped through the column until no more stationary phase eluted. Subsequently, a sample (60 mg/mL, dissolved in 5 mL of the upper and lower phases each) was injected. All separations were manually fractionated at 1-min intervals and monitored at 320 nm. The collected fractions were analyzed using HPLC-DAD. Those containing high-purity substances were combined and evaporated to dryness.

### 3.6. High-Performance Liquid Chromatography (HPLC-DAD)

HPLC-DAD was performed using a Shimadzu HPLC system (Shimadzu, Tokyo, Japan), equipped with a diode-array detector for peak detection. The mobile phase consisted of a water (A) and methanol (B) gradient system, and the stationary phase was a C18 column (5 µm, 250 × 4.6 mm). The following gradient was applied: 0.01 min—50% B; 5 min—60% B; 20 min—80% B; 25 min—100% B; 26 min—50% B; 35 min—50% B. The flow rate was maintained at 1 mL/min, with the column temperature set at 25 °C. The injection volume of the sample was 10 µL, and detection was carried out at 254 and 300 nm.

### 3.7. Identification

The chemical composition of *S. foliosum* seed and root MeOH and Et_2_O extracts was identified using the GS-MS technique. The full structural elucidation of CPC isolated compound was performed by HPLC-ESI-QTOF-MS/MS and 1D- and 2D-nuclear magnetic resonance (NMR).

### 3.8. HPLC-ESI-QTOF-MS/MS

HPLC-ESI-QTOF-MS/MS analysis was conducted using an Agilent 1200 HPLC system (Agilent Technologies, Santa Clara, CA, USA). The system was equipped with a degasser (G1379B), a binary pump (G1312C), a thermostated column compartment (G1316A), an auto-sampler (G1329B), a diode array detector (G1315D) and ESI-QTOF mass spectrometer (G6530B). The separations were carried out on a Phenomenex (Torrance, CA, USA) Gemini C18 (3 um, 100 × 2 mm) column with a mobile phase consisting of 0.1% formic acid + 10 mM ammonium formate (pH = 3.5) + 1% acetonitrile in water (solvent A) and 0.1% formic acid + 10 mM ammonium formate (pH = 3.5) + 95% acetonitrile in water (solvent B). The following gradient was applied: 0–10 min: 40% B; 30–35 min: 80% B; 40–45 min: 100% B. The following MS parameters were used: positive ionization mode; mass range: 100–1000 *m*/*z*; gas temperature 350 °C; nitrogen flow: 10 L/min; nebulizer pressure 40 psi; skimmer 65 V; capillary voltage 3500 V; fragmentor 100 V; collision-induced dissociation energy 10 V [12]. Identification of the compounds was performed based on mass to charge ratio measurements (*m*/*z*) of molecular ions and fragments in MS/MS experiments. On this basis, the fragmentation patterns was obtained which was compare with databases (METLIN, Massbank, GNPS) and literature data.

### 3.9. Nuclear Magnetic Resonance Spectroscopy (NMR)

The 1D and 2D NMR spectra (^1^H, ^13^C DEPTQ, HSQC, HMBC, ^1^H–^1^H COSY DQF, NOESY) were recorded on a Bruker Avance III HD Ascend-500 spectrometer (Bruker BioSpin, Rheinstetten, Germany), equipped with a 5 mm ^1^H{^109^Ag–^31^P} broad-band inverse (BBI) probe at 30 °C in deuterated methanol-*d4* (99.96% D).

### 3.10. Gas Chromatography with Mass Spectrometry (GC-MS)

The GC-MS was used to examine and identify the volatile components of the extract. The instrument was Agilent Technologies 7000 GC/MS Triple Quad. The separations were carried out on the Elite 5-MS; 30 m × 250 μm × 0.25 μm column. The mass range was between 40 and 330 *m*/*z*, and the ionization energy was 70 eV, scanning mode: Total Ion Current (TIC), scanning range: 40 to 600 Da. The following parameters were applied: initial oven temperature: 40 °C for 4 min; Ramp 1: 40 °C to 250 °C at 10°C/min, hold for 2 min; Ramp 2: 250 °C to 300 °C at 15 °C/min, hold for 5 min. Injector temperature: 250 °C, injection volume: 1 µL, transfer line temperature: 300 °C, carrier gas: helium. The flow rate was set to 1 mL/min, and the split mode was chosen (split ratio 1:10 or 1:100).

The mass spectrometric settings were full scan mode, 20,000 amu/s scan speed, and 50 spectra per sample frequency. Temperatures at the contact and ion source were 250 °C and 200 °C, etc. Volatile component identities were confirmed through spectral matching with reference standards where available, as well as with the spectral libraries of the National Institute of Standards and Technology (NIST05a), Flavor, and MassFinder 4. In addition, the retention indices (RI) obtained using the C8–C20 alkane range as reference points, were compared to literature values [66].

### 3.11. Evaluation of Total Phenolic Content

Total phenolics were quantified according to the procedures outlined by Uysal et al. [67]. Briefly, 50 µL aliquot of the sample solution was mixed with 100 µL of diluted Folin–Ciocalteu reagent (1:9, *v*/*v*) and shaken vigorously. After 3 min, 75 µL of 1% Na₂CO₃ solution was added, and the mixture was incubated at room temperature for 2 h. Absorbance was measured at 760 nm, and the results were expressed as milligrams of gallic acid equivalents per gram of extract (mg GAE/g extract). The value of the calibration curve: absorbance = 0.268 (μg gallic acid) (R2: 0.9988, concentration range: 0–3 μg gallic acid).

### 3.12. Antioxidant Activity

Various antioxidant tests were carried out. The outcomes were represented as milligrams of Trolox equivalents (TE) per gram for the DPPH, ABTS radical scavenging, CUPRAC, and FRAP tests. In millimoles of TE per gram of extract, the phosphomolybdenum (PBD) test examined antioxidant potential, and in milligrams of disodium edetate equivalents (EDTAE) per gram of extract, the metal chelating activity (MCA) was determined. The methods were described by Uysal et al. [67] and Grochowski et al. [68].

### 3.13. Anti-Enzymatic Activity

Experiments on enzyme inhibition were performed on the samples. Acarbose equivalents (ACAE) per gram of extract was used to measure the activities that inhibit amylase and glucosidase, while milligrams of galanthamine equivalents (GALAE) per gram of extract was used to examine the inhibition of AChE and BChE. The amount of tyrosinase inhibition for each gram of extract was measured in milligrams of kojic acid equivalents (KAE). The details were given in our previous study [68].

### 3.14. Microbiological Studies

#### 3.14.1. Minimal Inhibitory Concentration (MIC)

The antibacterial and antifungal activities were screened for *S. foliosum* root and seed methanolic extracts by microdilution broth method according to the European Committee on Antimicrobial Susceptibility Testing (EUCAST) using Mueller–Hinton broth and MH with 2% glucose for growth of fungi. The extracts dissolved in DMSO were first diluted to the concentration (4000 µg/mL) in an appropriate broth medium recommended for bacteria or yeasts. Then, using the same media, serial two-fold dilutions were made in order to obtain final concentrations of the tested derivatives ranging from 4000 to 7.8 µg/mL. Sterile 96-well polystyrene microtiter plates (Nunc, Denmark) were prepared by dispensing 100 µL of the appropriately diluted tested extracts in broth medium into each well. The inocula were prepared by diluting fresh microbial cultures in sterile 0.85% NaCl to match a turbidity of 0.5 McFarland standard and were added to wells to obtain the final density of 5 × 10^5^ CFU/mL for bacteria and 5 × 10^4^ CFU/mL for yeasts; CFU—colony forming units. After incubation at 35 °C for 18–20 h, the minimum inhibitory concentrations (MICs) were determined both visually and spectroscopically by measuring absorbance at 600 nm. The MIC was defined as the lowest concentration of the extracts that inhibited the growth of the reference microbial strains. Appropriate controls were included in each experiment: a DMSO control (final concentration of 10%, *v*/*v*), a positive control (inoculum without the tested derivatives), and a negative control (tested derivatives without inoculum). Vancomycin, ciprofloxacin, and nystatin were used as the reference drugs for Gram-positives, Gram-negatives, and yeasts, respectively.

#### 3.14.2. Minimal Bactericidal and Fungicidal Concentration (MBC/MFC)

The minimal bactericidal concentration (MBC) or fungicidal concentration (MFC) was assessed by transferring 5 µL of the microbial culture from each well onto the appropriate agar plates. Following incubation at 35 °C for 24 h. The lowest broth dilution of extract that prevents the growth of the microorganism on the extract-free agar plate was reported as biocidal. The MBC/MFC was defined as the lowest concentration of the extracts that completely inhibited microbial growth. All experiments were conducted in triplicate, and the highest observed value was recorded.

### 3.15. Cytotoxicity and Anticancer Selectivity

The *S. foliosum* extracts were dissolved (50 mg/mL) in DMSO (cell-culture grade purity, PanReac Applichem) to obtain the stock solution for cytotoxicity evaluation. The cytotoxicity was evaluated using the previously described MTT-test methodology [69]. Briefly, the cells were passaged into 96-well plates and, after overnight incubation, treated with serial dilutions (1000–1 µg/mL) of tested extracts in cell media for 72 h. Afterwards, cell media was removed, the monolayers were washed with PBS, and MTT-supplemented media was added, and after 3 h the formazan product was dissolved using SDS/DMF/PBS solvent. Following overnight incubation, the Synergy H1 Multi-Mode Microplate Reader (BioTek Instruments, Inc. Vermont, U.S.) was used to measure the absorbance (540 and 620 nm), and collected data was exported to GraphPad Prism (version 9.0.0) to calculate the CC_50_ values (50% cytotoxic concentration) from dose–response curves (non-linear regression). Finally, based on the CC_50_ values, the selectivity indexes (SI) were calculated (SI = CC_50_Vero/CC_50_Cancer, SI > 1 suggests anticancer selectivity) to assess the specificity towards cancer cell lines.

### 3.16. Zebrafish Model for Evaluation Anxiety-Like Behaviors

#### 3.16.1. Zebrafish Husbandry

Zebrafish (*Danio rerio*) stocks of the wild type (AB strain, Experimental Medicine Centre, Medical University of Lublin, Poland) were maintained at 28 ± 0.5 °C on a 14/10 h light/dark cycle under standard aquaculture conditions, and fertilized eggs were collected via natural spawning. Embryos were reared under 14/10 h light/dark cycle in embryo medium: 1.5 mM, pH 7.1–7.3, 17.4 mM NaCl, 0.21 mM KCl, 0.12 mM MgSO_4_, and 0.18 mM Ca(NO_3_)_2_ at 28.5 °C. 5 days post-fertilization (dpf) were used for all experiments.

#### 3.16.2. Determination of the Maximum Tolerated Concentration

A stock solution of 50 mg/mL of extracts was prepared by dissolving in DMSO, and diazepam was prepared in concentration of 20 mM and stored in −20 °C prior to experiment.

For determination of the maximal tolerated concentration (MTC) 5-dpf larvae were placed in 48 well plates; 5 larvae in each well, 10 larvae for concentration. Extracts were tested in 4 different concentrations (100, 50, 25, and 10 µg/mL). After 18 h incubation in a dark environment, each larva was checked under the microscope for signs of acute locomotor impairment: weak response upon a light touch of the tail with a fine needle, loss of posture, body deformation, bulging of the eyes out of their sockets, slow or absent heartbeat, and death [70].

#### 3.16.3. Anxiolytic Activity Assay

The protocol was adopted after Banono et al. [71] with several modifications. For the measurement of locomotor activity, EthoVision (Noldus, Netherlands) software was used. On the day of the experiment, larvae were placed on a 24-well plate; single larvae per well, 6 larvae per concentration. After 30 min of incubation, the tested extracts in 3 different concentration plates were transferred from the incubator to the Noldus set-up and acclimated for 5 min in dark conditions under a temperature-controlled setting (27 ± 1 °C). This step was was followed by measurement of locomotor activity and light–dark transition for 10 min each of tracking in (1) darkness (0% light) (2) 100% light, and (3) darkness. For thigmotaxis, the area of 24 wells was divided by the outer and inner area (diameter of inner zone = 8 mm, inner zone distance in relation to the outer zone = 4 mm) and calculated using the following formula [72]: thigmotaxis (% distance in the outer zone) = ((distance in the outer zone)**/**(distance in outer zone + distance in the inner zone) × 100%.

## 4. Conclusions

This study provides a comprehensive analysis of the chemical composition and biological activities of *S. foliosum* extracts, underscoring its potential as a valuable source of natural bioactive compounds. The findings expand the existing knowledge of *S. foliosum* by detailing the substantial presence of monoterpenoids, sesquiterpenoids, phenolic compounds, and other secondary metabolites. The multifaceted investigation revealed mild antimicrobial activity, particularly against *S. aureus* and *C. glabrata*, strong antioxidant capacity, enzyme inhibitory properties, and cytotoxic effects against hypopharyngeal carcinoma cells. These results suggest that *S. foliosum* holds promise for the development of natural products with medicinal, agricultural, and industrial applications. Future research should focus on further exploring the mechanisms of action of these compounds, optimizing extraction and purification processes, and conducting in vivo studies to fully realize the potential of *S. foliosum* in clinical settings. This study paves the way for the development of *S. foliosum*-based products with broad-spectrum applications in pharmacology, healthcare, and beyond.

## Figures and Tables

**Table 1:** Volatile compounds of *Seseli foliosum* root and seed MeOH and Et_2_O extracts.

Volatiles	RT	RI	RI (*lit*)	MeOH ext.	Et_2_O ext.
Linalool	5.86	1146	1148 [24]	+	-
*cis*-Verbenol	6.30	1145	1144 [25]	+	-
Isoborneol	6.45	1159	1158 [25]	+	-
*α*-Pinocarvone	6.48	1160	1162 [25]	-	+
Safranal	6.50	1192	1193 [26]	+	-
Verbenone	6.80	1205	1206 [25]	+	+
p-Cymen-7-ol	7.09	1286	1287 [25]	-	-
(−)-Bornyl acetate	7.33	1282	1285 [25]	+	-
*α*-ylangene	8.0	1373	1372 [25]	+	-
Lauraldehyde	8.14	1405	1408 [25]	-	+
*α*-Bergamotene	8.35	1412	1415 [25]	+	-
Caryophyllene	8.40	1423	1424 [27]	+	-
*β*-Bisabolene	8.80	1508	1509 [25]	+	-
Globulol	8.82	1582	1583 [25]	-	+
(−)-Spathulenol	9.06	1574	1576 [25]	+	+
Caryophyllene oxide	9.12	1579	1581 [25]	+	+
Epiglobulol	9.80	1582	1585 [25]	+	-
Palmitic acid	10.97	1965	1968 [25]	-	+
Phytol	11.72	2113	2114 [25]	-	+
Linoleic acid	11.81	2129	2130 [25]	-	+
Oleic acid	12.12	2138	2137 [25]	+	-

**Table 2:** Total content of phenolic compounds.

Extract	TPC (mg GAE/g)
SfSMeOH	19.27 ± 0.38
SfRMeOH	10.41 ± 0.10

**Table 3:** Determination of the antioxidant activity of SfSMeOH and SfRMeOH extracts. (Description: TE/g—trolox equivalent, EDTAE/g—ethylendiaminetetra acetic acid equivalent, CUPRAC—cupric ion reducing activity; FRAP—ferric reducing antioxidant power; MCA—metal chelating activity; PDA—phosphomolybdenum method).

Extract	DPPH (mg TE/g)	ABTS (mg TE/g)	CUPRAC (mg TE/g)	FRAP (mg TE/g)	MCA (mg EDTAE/g)	PDA (mmol TE/g)
SfSMeOH	37.97 ± 0.90	28.94 ± 0.22	56.18 ± 2.47	35.86 ± 0.85	27.51 ± 0.29	1.19 ± 0.07
SfRMeOH	10.76 ± 0.19	14.12 ± 0.24	28.87 ± 0.59	16.05 ± 0.69	19.97 ± 2.09	1.23 ± 0.09

**Table 4:** Enzyme inhibitory effect of *S. foliosum* seed and root MeOH extracts.

Extract	ACHE (mg GALAE/g)	BCHE (mg GALAE/g)	Tyrosinase (mg KAE/g)	α-Amylase (mg ACAE/g)	α-Glucosidase (mg ACAE/g)
SfSMeOH	2.70 ± 0.11	2.27 ± 0.34	36.74 ± 1.03	0.47 ± 0.03	0.73 ± 0.1
SfRMeOH	2.79 ± 0.13	1.73 ± 0.28	28.98 ± 1.08	0.45 ± 0.01	0.72 ± 0.01

**Table 5:** MIC and MBC values (µg/mL) of *S. foliosum* seed (SfSMeOH) and root (SfRMeOH) MeOH, *S. foliosum* aerial (SfSV) and under-ground (SfRV) diethyl ether extracts against reference bacterial and fungal strains (NA, not applicable).

Microorganisms	SfSMeOH	SfRMeOH	SfSV	SfRV	Reference Antibiotics
**Gram-positive bacteria**					Vancomycin
*Staphylococcus aureus* ATCC 25923	125	250	250	1000	0.98
*Staphylococcus epidermidis* ATCC 12228	250	250	2000	4000	0.98
*Micrococcus luteus* ATCC 10240	125	62.5	1000	250	0.12
*Enterococcus faecalis* ATCC 29212	>4000	>4000	2000	4000	1.95
*Bacillus subtilis* ATCC 6633	250	500	2000	4000	0.24
*Bacillus cereus* ATCC 10876	250	125	>4000	1000	0.98
**Gram-negative bacteria**					Ciprofloxacin
*Salmonella* Typhimurium ATCC 14028	>4000	>4000	>4000	4000	0.061
*Escherichia coli* ATCC 25922	>4000	>4000	>4000	4000	0.015
*Proteus mirabilis* ATCC 12453	>4000	>4000	2000	4000	0.030
*Klebsiella pneumoniae* ATCC 13883	>4000	>4000	>4000	4000	0.122
*Pseudomonas aeruginosa* ATCC 9027	>4000	>4000	>4000	4000	0.488
**Fungi**					Nystatin
*Candida albicans* ATCC 2091	2000	>4000	2000	>4000	0.48
*Candida parapsilosis* ATCC 22019	500	>4000	2000	>4000	0.24
*Candida glabrata* ATCC 90030	62.5	>4000	4000	>4000	0.24

**Table 6:** The CC_50_ obtained for *S. foliosum* methanolic extracts and volatiles on a panel of cell lines.

*S. foliosum*	VERO	AGS	FaDu	RKO
SfSMeOH	440.40 ± 37.48 *	242.43 ± 20.98	186.80 ± 12.73	151.10 ± 3.35
SfRMeOH	320.60 ± 32.10	264.23 ± 14.70	135.33 ± 6.10	119.07 ± 14.03
SfSV	133.90 ± 11.11	151.70 ± 10.57	22.33 ± 0.47	41.69 ± 1.32
SfRV	213.77 ± 1.96	132.90 ± 1.97	27.16 ± 2.19	51.18 ± 4.18

* CC_50_, 50% cytotoxic concentration; mean ± SD (*n* ≥ 3), µg/mL.

## Data Availability

The original contributions presented in this study are included in the article/Supplementary Materials. Further inquiries can be directed to the corresponding author.

## Supplementary Materials

The following supporting information can be downloaded at: https://www.mdpi.com/article/10.3390/molecules30030725/s1. Table S1: ^1^H- and ^13^C-NMR special data (500 MHz, *J* in Hz, in CD_3_OD); Figure S1: HPLC-DAD chromatogram of *Seseli foliosum* seed MeOH (SfSMeOH) extract; Figure S2: ESI-QTOF-MS spectrum of edultin.
