# Supplementary material for: Seseli foliosum (Somm. et Levier) Manden.—A Comprehensive Phytochemical and Biological Evaluation
Source: Molecules. 2025 Feb 5;30(3):725. doi: 10.3390/molecules30030725 (PMC11820956; doi:10.3390/molecules30030725)
Supplement: Supplementary file 1 [file molecules-30-00725-s001.zip › molecules-3429288-supplementary.pdf]

Supplementary materials

Seseli foliosum (Somm. et Levier) Manden. — A comprehensive Phytochemical and Biological Evaluation

Mariam Nersezashvili [1], Dali Berashvili [1], Malkhaz Jokhadze [1], Mariam Metreveli [2], Łukasz Świątek [3], Kinga Salwa [3], Adrianna Skiba [4], Łukasz Pecio [4,5], Krzysztof Kamil Wojtanowski [6], Izabela Korona-Głowniak [7], Gökhan Zengin [8], and Krystyna Skalicka-Woźniak [4,*]

[1] Tbilisi State Medical University, Faculty of Pharmacy, Direction of Pharmacognosy and Pharmaceutical Botany, Tbilisi 0186, Georgia; m.nersezashvili@tsmu.edu; d.berashvili@tsmu.edu; m.jokhadze@tsmu.edu
[2] Batumi Shota Rustaveli State University, Institute of Phytopathology and Biodiversity, Batumi 6010, Georgia; metreveli.mariam@bsu.edu.ge
[3] Medical University of Lublin, Department of Virology with Viral Diagnostics Laboratory, Lublin 20-093, Poland; lukasz.swiatek@umlub.pl; kinga.salwa@umlub.pl
[4] Medical University of Lublin, Department of Natural Products Chemistry, Lublin 20-093, Poland; lukasz.pecio@umlub.pl; adrianna.skiba@umlub.pl; kskalicka@pharmacognosy.org
[5] Department of Phytochemistry, Institute of Soil Science and Plant Cultivation – State Research Institute, 24-100 Puławy, ul. Czartoryskich 8, Poland
[6] Medical University of Lublin, Department of Pharmacognosy with Medicinal Plant Garden, Lublin 20-093, Poland; krzysztofkamilw@gmail.com
[7] Medical University of Lublin, Department of Pharmaceutical Microbiology, Lublin 20-093, Poland; izabela.korona-glowniak@umlub.pl
[8] Department of Biology, Faculty of Science, Selcuk University, Konya 42130, Turkey; gokhanzengin@selcuk.edu.tr
* Correspondence: kskalicka@pharmacognosy.org ; Tel.: +48 81448 7086

Table S1. ^{1}H- and ^{13}C-NMR spectral data (500 MHz, *J* in Hz, in CD_3OD)

Position	^{1}H	^{13}C
**2**	-	161.8
**3**	6.23 (1H, d, *J* = 9.6)	113.5
**4**	7.89 (1H, d, *J* = 9.6)	145.9
**4a**	-	115.0
**5**	7.62 (1H, d, *J* = 8.5)	133.3
**6**	6.94 (1H, d, *J* = 8.5)	108.8
**7**	-	165.1
**8**	-	113.8
**8a**	-	152.9
**2′**	5.32 (1H, d, *J* = 6.8)	89.8
**3′**	7.07 (1H, d, *J* = 6.8)	69.7
**4′**	-	82.2
**4′ *gem*-Me**		
	1.72 (3H, s)	25.7
	1.60 (3H, s)	22.9
**-*O*-Ac**		
**1**	-	172.2
**2**	1.98 (3H, s)	22.3

**-*O*-Angeloyl**		
**1**	-	167.6
**2**	-	128.6
**3**	6.13 (1H, qq, *J* = 7.2, 1.6)	139.8
**4**	1.91 (3H, dq, *J* = 7.2, 1.6)	15.8
**2-Me**	1.83 (3H, q, *J* = 1.6)	20.6

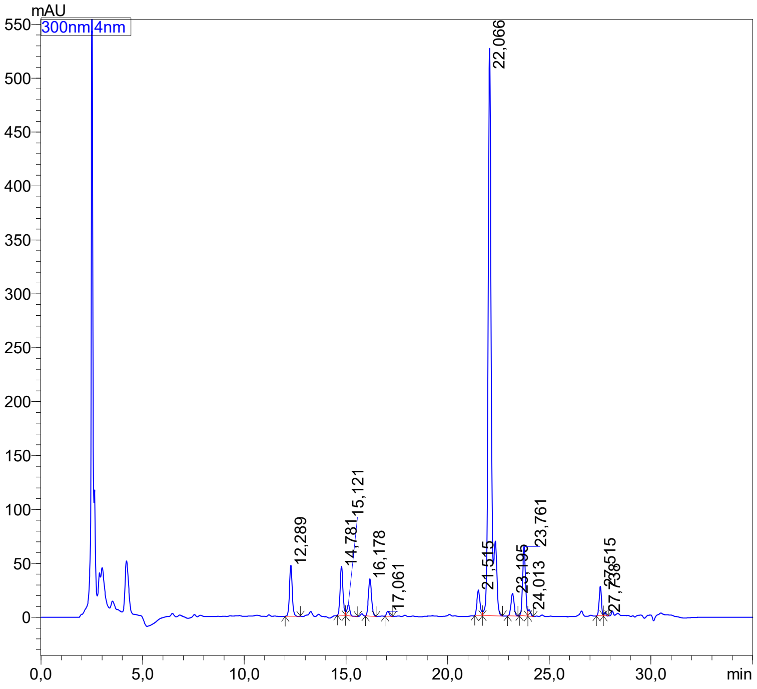

Figure S1. HPLC-DAD chromatogram of *Seseli foliosum* seed MeOH (SfSMeOH) extract

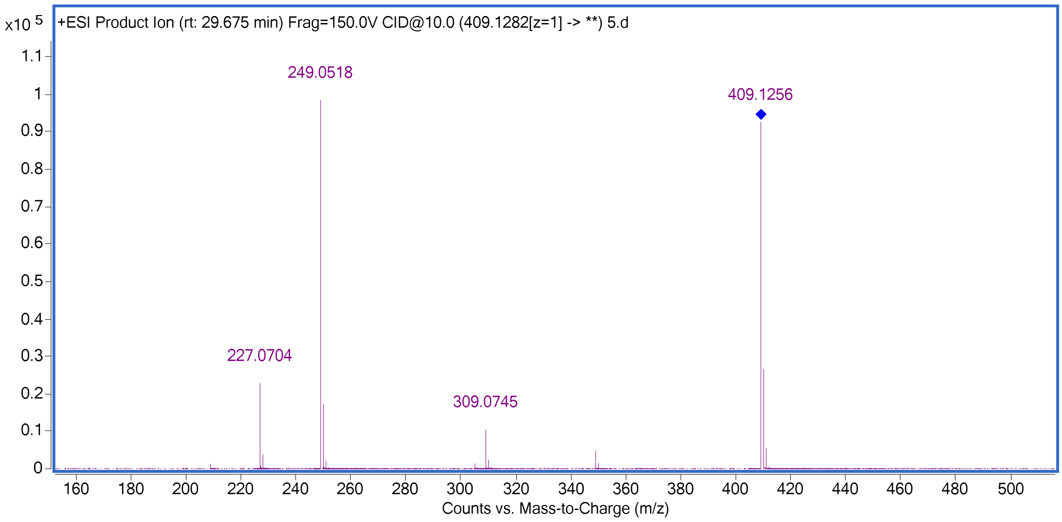

Figure S2. ESI-QTOF-MS spectrum of edultin

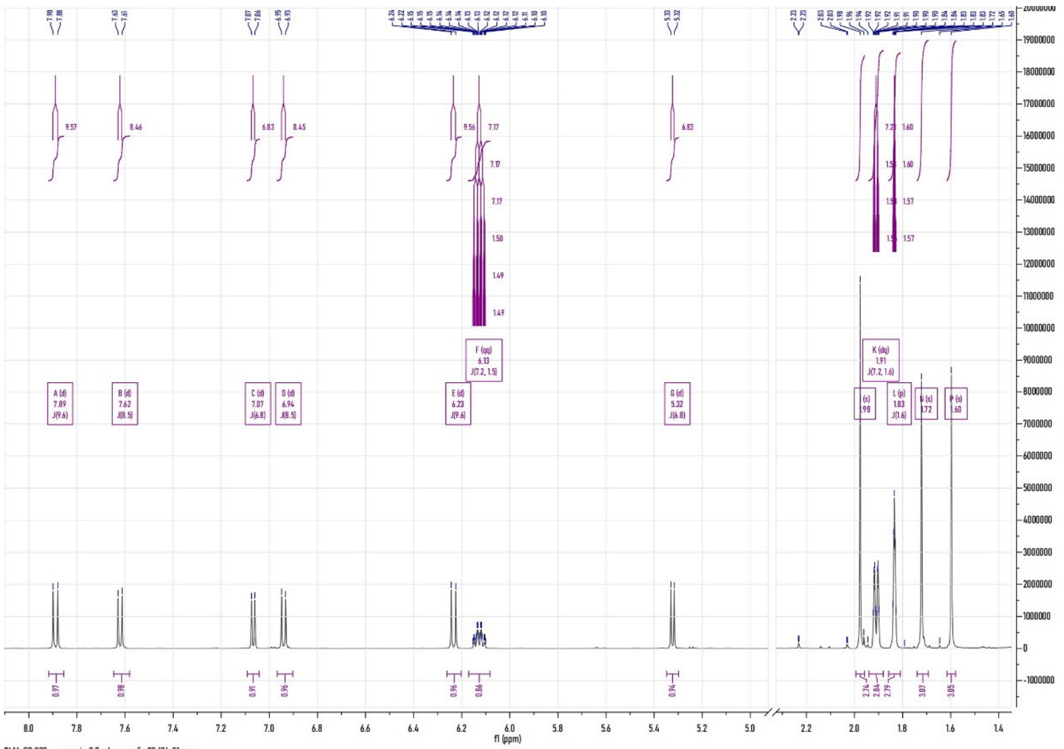

a. 1H MeOD 30C — x mg in 0.7 mL — sep5.p20 f26-31 s.s.

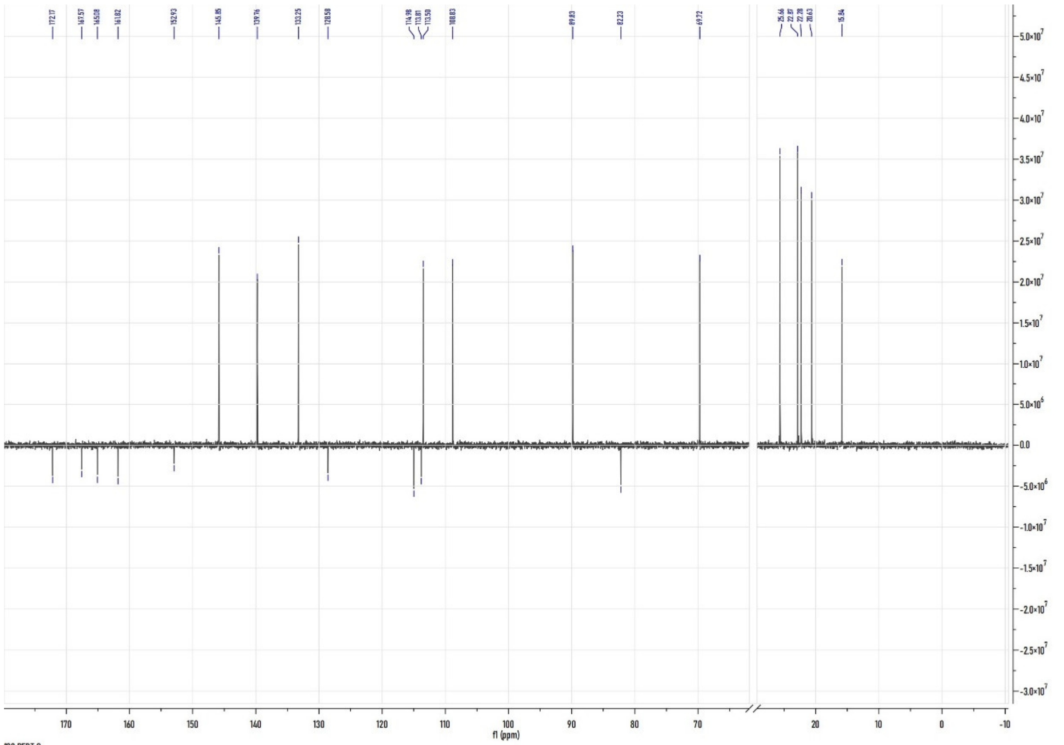

b. 13C DEPT Q

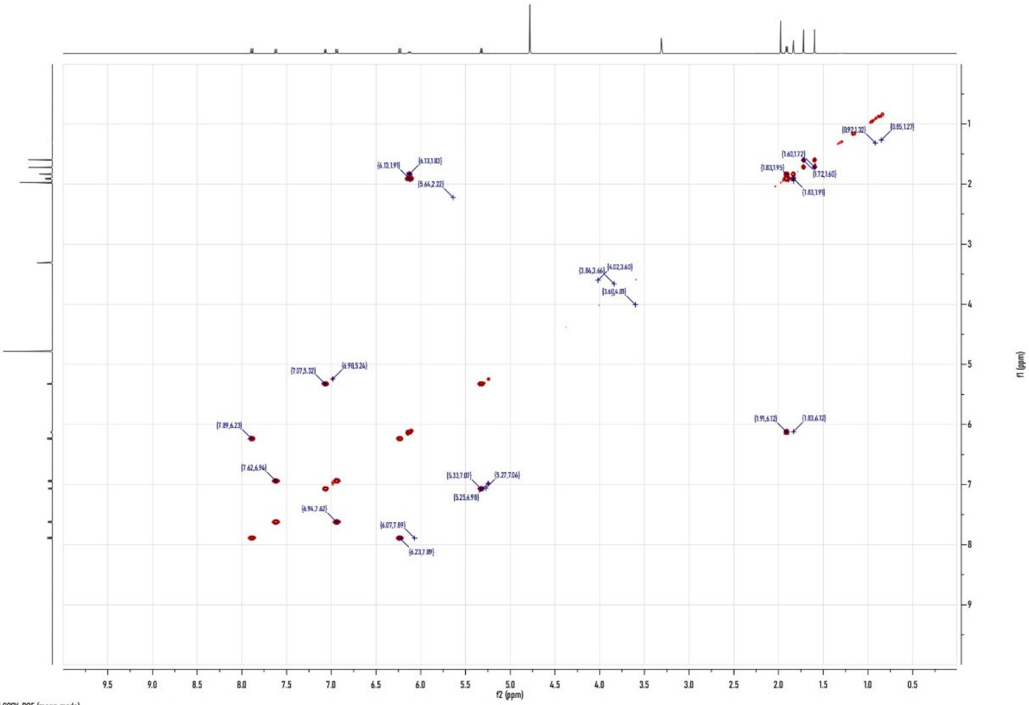

c. 2D COSY-DQF (magn mode)

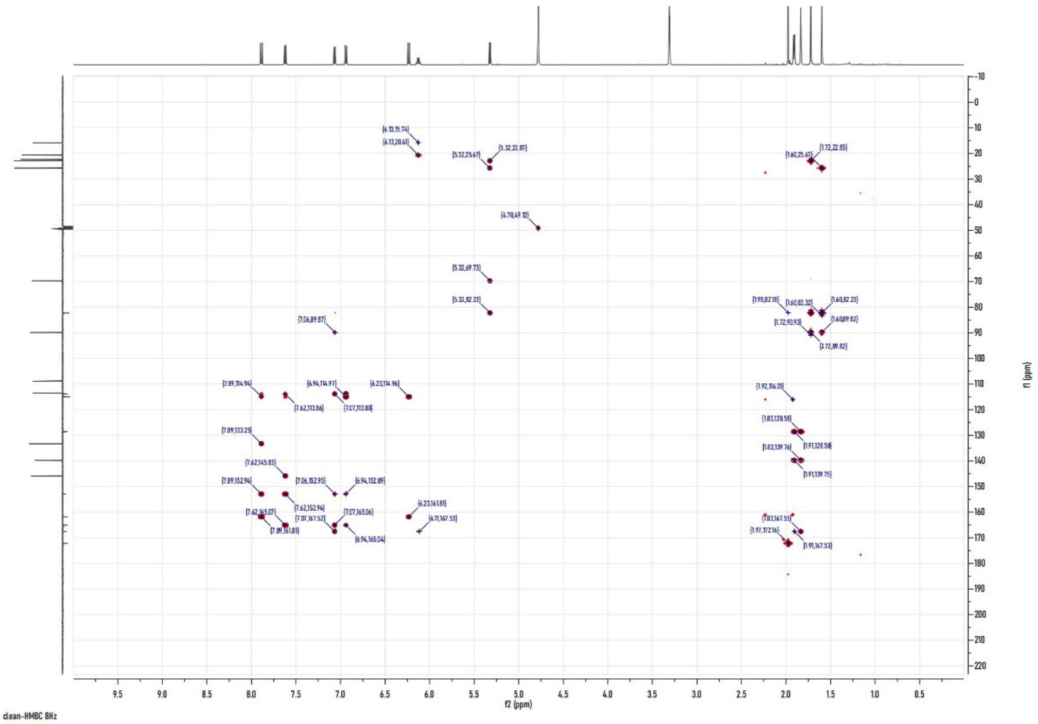

d. clean-HMBC 8Hz

e.

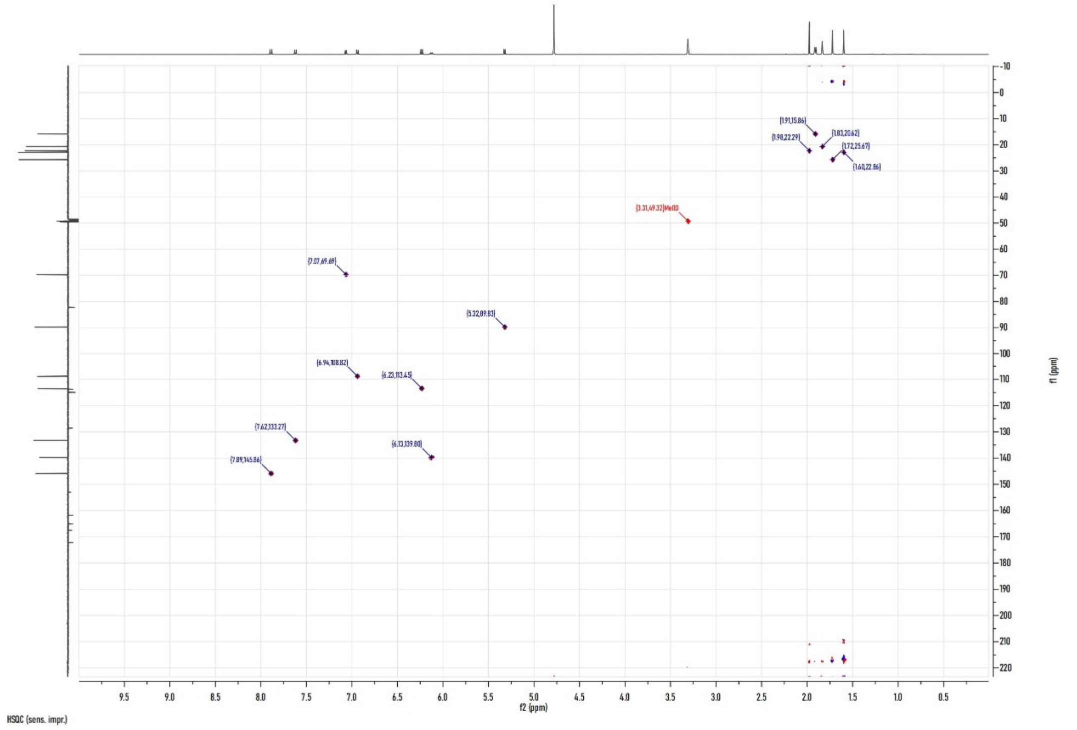

f.

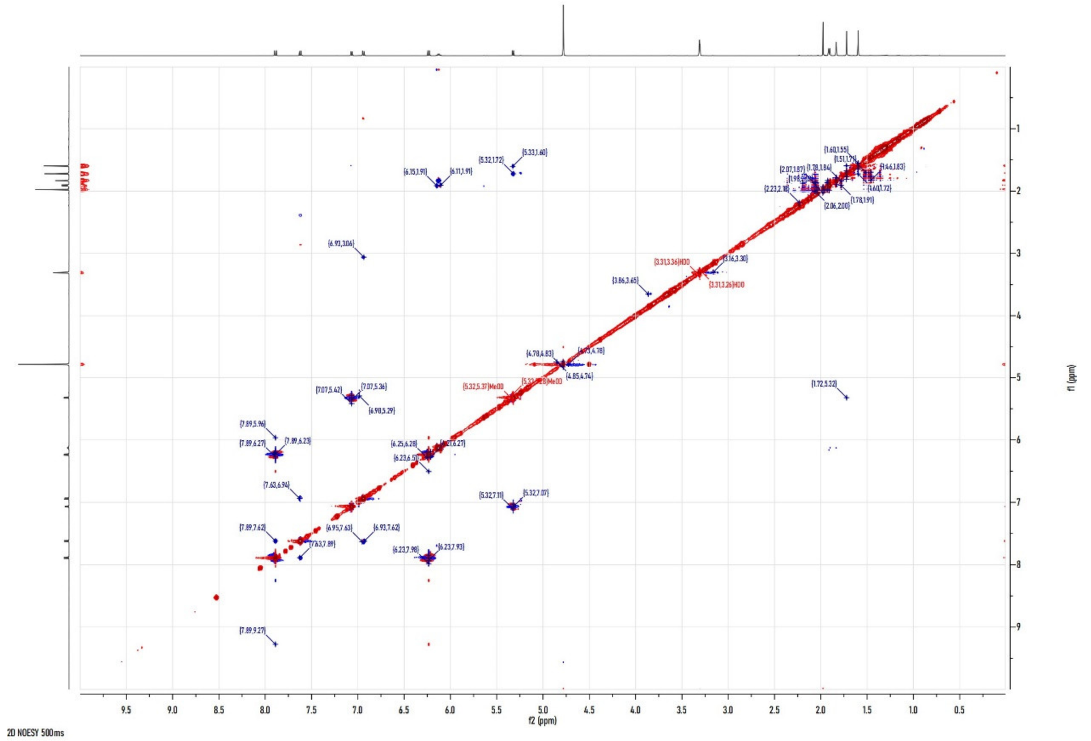

Figure S3. NMR spectra of the isolated compound - edultin, in MeOH-d_4, recorded at 30°C on a 500 MHz instrument: a) ^{1}H; b) ^{13}C DEPTQ-135; c) ^{1}H-^{1}H COSY *double-quantum-filtered*; d) ^{1}H-^{13}C HMBC; e) ^{1}H-^{13}C HSQC; f) ^{1}H-^{1}H NOESY.
